# CTLA-4 and Autoimmunity: New Twists in the Tale

**DOI:** 10.1016/j.it.2015.11.002

**Published:** 2015-12

**Authors:** Lucy S.K. Walker

**Affiliations:** 1 Institute for Immunity & Transplantation, University College London Division of Infection & Immunity, Royal Free Campus, London, NW3 2PF, UK

## Abstract

CTLA-4 has long been associated with control of autoimmunity. A recent study by Sharpe and colleagues explores this relationship in a model that enables conditional deletion of CTLA-4 in adult mice, with some surprising new conclusions.

CTLA-4 is one of a small number of immune genes whose deletion triggers fatal lymphoproliferative disease. Its importance in immune regulation is unquestioned; however, its precise mechanism of action has been hotly debated. In a recent paper in *The Journal of Experimental Medicine* [1] the Sharpe laboratory, who originally reported the phenotype of CTLA-4 null mice, brings us another intriguing installment in the CTLA-4 story. By generating mice with a floxed CTLA-4 allele, and crossing them to mice expressing a tamoxifen inducible Cre-recombinase, the authors constructed an experimental system in which CTLA-4 could be ablated ‘at will’ by tamoxifen administration. This permits for the first time the deletion of the CTLA-4 gene only in adult mice.

The first surprise in the data is that punctual ablation of the CTLA-4 gene in 7-week old mice failed to trigger spontaneous autoimmunity, contrasting with the situation following germline deletion. Furthermore, the CTLA-4-deleted mice exhibited reduced susceptibility to MOG-induced experimental autoimmune encephalomyelitis (EAE) and did not exhibit an increased capacity to clear tumors, both opposite to expectations based on the concept that CTLA-4 inhibits autoimmunity and limits anti-tumor responses.

So what is the basis for these striking findings? By breeding the tamoxifen deletable CTLA-4 mice to a MOG-specific TCR transgenic mouse (2D2) the authors first homed in on the role of CTLA-4 in conventional T (Tconv) cells. Adoptively transferred 2D2 T cells induced EAE in RAG-deficient mice with similar onset and severity regardless of whether these cells expressed CTLA-4 or not. An elegant approach in which CTLA-4 was deleted from IL-17 expressing cells T cells (IL-17F-Cre) also revealed no difference in EAE incidence. Next, adoptive transfer experiments were performed in which both T regulatory (Treg) cells and Tconv cells were injected into recipient mice, but CTLA-4 deficiency was restricted to the latter: these experiments revealed a subtle increase in Tconv cell number in the absence of CTLA-4, although proliferative responses to MOG peptide or anti-CD3 and cytokine differentiation *in vitro* were not altered. Thus, CTLA-4 deficiency in the Tconv compartment had small but discernable biological consequences; however, it did not alter EAE induction.

By contrast, deletion of CTLA-4 from the Treg compartment was sufficient to recapitulate the effects of systemic CTLA-4 deletion, with similar protection from EAE being observed. Thus in keeping with many other experimental settings, the major role of CTLA-4 was shown to be in Treg cells; however, unexpectedly this resulted in disease protection rather than disease exacerbation.

In keeping with previous work, the authors observed a dramatic expansion of the Treg cell population in CTLA-4-deleted mice, reflecting a marked increase in their proliferation (∼60% Treg cells were Ki67^+^ in CTLA-4-deleted mice as compared to ∼30% in controls) [1]. This nicely parallels the increased Treg cell proliferation seen in mice lacking CTLA-4 from birth (∼62% Treg were Ki67^+^ in CTLA-4^−/−^ mice compared with ∼28% in littermate controls) [2]. By ablating CTLA-4 specifically in the Treg compartment, using a tamoxifen-inducible cre under the control of the Foxp3 promoter, the authors were able to confirm that the Treg cell expansion was a direct effect of CTLA-4 in the Treg cells themselves. As the authors surmise, this Treg cell expansion following CTLA-4 ablation likely reflects unrestrained CD28 signaling, since CTLA-4 is known to compete with CD28 for access to their shared ligands CD80 and CD86.

It has been shown previously that CTLA-4^−/−^ Treg cells can exhibit a compensatory overproduction of both IL-10 and TGF-β [3]. Indeed, the Powrie group demonstrated that while suppression of colitis by wild type Treg cells was CTLA-4 dependent, CTLA-4^−/−^ Treg cells were able to use IL-10 to elicit suppression [4], providing a precedent for the compensation of CTLA-4 function by the IL-10 pathway. Data presented by Paterson and colleagues suggest that a similar situation may arise following inducible deletion of CTLA-4: In the context of the EAE model, CTLA-4 ablation was associated with a marked increase in the proportion of Treg cells producing IL-10. The functional impact of this could be potentiated by the greater than 3 fold increase in the absolute numbers of Treg cells. Thus, although most of these Treg cells could no longer use CTLA-4, the population was expanded in number and had an enhanced capacity to produce IL-10 (Figure 1). NanoString transcriptional analysis confirmed that the Treg cells adopted a gene expression signature associated with high IL-10 production, including increased expression of the transcription factor Blimp-1. Therefore, one explanation for the paradoxical disease protection following CTLA-4 ablation could be the altered cytokine environment imposed by an expanded population of Treg cells that overproduce IL-10.

The authors next set out to test whether CTLA-4- ablated Treg cells had the capacity to elicit suppressive function. They first employed *in vitro* suppression assays, and demonstrated equivalent suppressive function regardless of whether the Treg cells derived from tamoxifen-treated (hence CTLA-4-ablated) animals. This result is not entirely unexpected, since several groups have found that CTLA-4^−/−^ Treg cells exhibit intact suppression in such *in vitro* assays 2, 3, 5. Moving to an *in vivo* setting, the authors went on to show that CTLA-4-ablated Treg cells retained the capacity to control the homeostatic proliferation of T cells adoptively transferred to Rag-deficient recipients. This contrasts with the findings of Sojka *et al*. [5] who showed that CTLA-4^−/−^ Treg were impaired in their capacity to control lymphopenia-induced T cell expansion. This could reflect a differential requirement for CTLA-4 in particular Treg cell subsets – Sojka *et al.* focused on CD62L^+^Treg cells. Alternatively, it might reflect residual CTLA-4 activity resulting from incomplete gene deletion in the tamoxifen system.

Curiously, despite conferring protection from EAE, loss of CTLA-4 from Treg cells triggered a significant expansion of Tconv cells in the cervical lymph nodes, with more cells expressing ki67 and bearing a CD44^hi^CD62L^lo^ phenotype, consistent with a role for Treg cells in suppressing Tconv cell activation via CTLA-4. However, if Tconv cell responses are unleashed following Treg cell CTLA-4 ablation, why then is this associated with disease protection? One possibility is that although Tconv cells become activated, they differentiate in a manner that leads them to be non-pathogenic, at least in EAE. Alterations in T cell cytokine production are known to affect the disease course in the EAE model, sometimes in unexpected ways. For example increases in one cytokine (e.g., IFNγ) can lead to decreases in another (e.g., IL-17) and consequent disease suppression. Paterson *et al*. showed that these Tconv cells exhibited higher levels of Ebi3, a subunit of the cytokine IL-27, which is known to inhibit Th17 differentiation and suppress EAE [6]. IL-27 is also recognized for its role in promoting the formation of type 1 regulatory T (Tr1) cells [6], and it is notable that amounts of several typical Tr1 products are increased in the Tconv cells (e.g., IL-10, Ahr, ICOS, LAG3). Thus one could envisage the expanded population of IL-10-producing Treg cells providing a favorable environment for induction of Tr1 cells that might in turn contribute to disease protection.

When the authors crossed the mice expressing a floxed CTLA-4 allele with mice bearing Foxp3-cre, this recapitulated the lethal phenotype reported by others [7], illustrating that CTLA-4 deficiency restricted to the Treg compartment is sufficient to cause fatal disease. Given that inducible CTLA-4 deletion in adult mice (in all cells or only in Treg cells) did not elicit disease, the authors suggest there may be a critical window of time developmentally during which CTLA-4 expression in Treg cells is essential. This could, for example, reflect a need for CTLA-4 during the neonatal period that diminishes once the peripheral immune compartment is fully seeded. Arguing against the need for CTLA-4 solely in the neonatal period is the observation that mixed bone marrow chimeras, comprising CTLA-4^−/−^ and wild type cells, rapidly become sick if the wild type cells are deleted during adulthood [8]. Such a hypothesis is also hard to reconcile with the studies that demonstrate defective function of CTLA-4^−/−^ Treg cells in adoptive transfer models, which typically use adult donors and recipients. A comprehensive comparison between conditional CTLA-4 deletion in neonates versus adult mice will be important in exploring this hypothesis further.

An alternative explanation for the lack of disease following ablation of CTLA-4 in adult mice could relate to the efficiency of tamoxifen-driven gene excision in Treg cells, Tconv cells and their precursors. For example, ∼ 7% of Treg cells still expressed CTLA-4 after tamoxifen treatment, and it is possible that these contribute to the observed lack of autoimmune disease. Incomplete deletion of CTLA-4 could potentially mirror the situation in humans with heterozygous CTLA-4 deficiency 9, 10, some of whom are asymptomatic. Intriguingly, quantitative defects in CTLA-4 in humans are associated with significant expansion of the Treg cell compartment [9], and it is tempting to speculate that protection from autoimmunity in asymptomatic individuals results from compensatory mechanisms such as the overproduction of IL-10 reported here. Notably the autoimmune phenotype in symptomatic individuals with CTLA-4 heterozygosity manifests relatively late in life, contrasting with the early onset associated with Foxp3 deficiency in IPEX. It will be of great interest to track the status of CTLA-4-ablated mice during aging and following a variety of immunological challenges to gain a broader perspective on the role of CTLA-4 in different contexts. Meanwhile, the increasingly refined tools being used to unravel the biology of this critical immune regulator will doubtless continue to turn up new surprises.

## Figures and Tables

**Figure 1:**
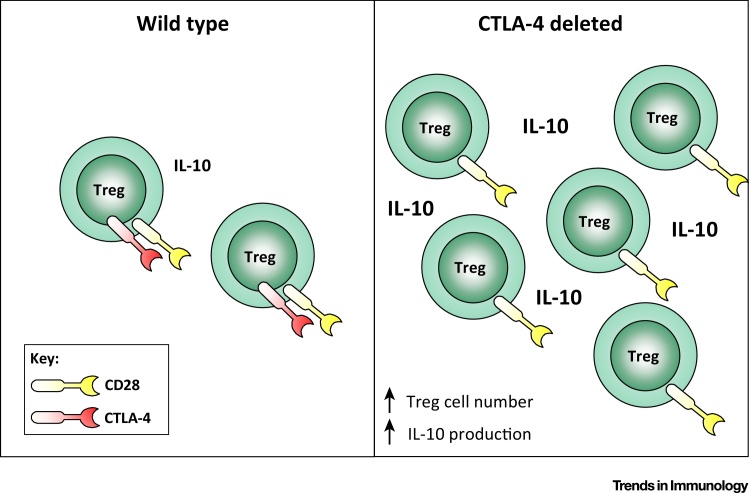
Punctual CTLA-4 Ablation Triggers an Expansion of Treg Cells that Overproduce IL-10. Deletion of CTLA-4 in adult mice, in all cells or specifically in Treg cells, is associated with an increase in Treg cell number and IL-10 production. The overabundance of IL-10 may suppress autoimmunity, perhaps via the induction of Tr1 cells.
